# Inhibition of Glioblastoma Growth by the Thiadiazolidinone Compound TDZD-8

**DOI:** 10.1371/journal.pone.0013879

**Published:** 2010-11-08

**Authors:** Diana Aguilar-Morante, Jose Angel Morales-Garcia, Marina Sanz-SanCristobal, Miguel Angel Garcia-Cabezas, Angel Santos, Ana Perez-Castillo

**Affiliations:** 1 Instituto de Investigaciones Biomédicas, Consejo Superior de Investigaciones Científicas-Universidad Autónoma de Madrid, and Centro de Investigación Biomédica en Red sobre Enfermedades neurodegenerativas (CIBERNED), Madrid, Spain; 2 Departamento de Bioquímica y Biología Molecular, Facultad de Medicina, Universidad Complutense de Madrid, Madrid, Spain; 3 Departamento de Anatomía Patológica, Hospital Universitario “La Paz”, Madrid, Spain; The University of Chicago, United States of America

## Abstract

**Background:**

Thiadiazolidinones (TDZD) are small heterocyclic compounds first described as non-ATP competitive inhibitors of glycogen synthase kinase 3β (GSK-3β). In this study, we analyzed the effects of 4-benzyl-2-methyl-1,2,4-thiadiazolidine-3,5-dione (TDZD-8), on murine GL261 cells growth *in vitro* and on the growth of established intracerebral murine gliomas *in vivo*.

**Methodology/Principal Findings:**

Our data show that TDZD-8 decreased proliferation and induced apoptosis of GL261 glioblastoma cells *in vitro*, delayed tumor growth *in vivo*, and augmented animal survival. These effects were associated with an early activation of extracellular signal-regulated kinase (ERK) pathway and increased expression of EGR-1 and p21 genes. Also, we observed a sustained activation of the ERK pathway, a concomitant phosphorylation and activation of ribosomal S6 kinase (p90RSK) and an inactivation of GSK-3β by phosphorylation at Ser 9. Finally, treatment of glioblastoma stem cells with TDZD-8 resulted in an inhibition of proliferation and self-renewal of these cells.

**Conclusions/Significance:**

Our results suggest that TDZD-8 uses a novel mechanism to target glioblastoma cells, and that malignant progenitor population could be a target of this compound.

## Introduction

Glioblastomas (GBM) are the most frequent and aggressive neoplasm among human primary brain tumors [1]. Despite many efforts to overcome this aggressive disease the median survival of patients with GBM remains less than 12 months from the time of diagnosis [1], [2]. Advances in glioma modeling in the mouse have made the disease amenable to *in vivo* functional and molecular studies [3]. Although there have been important advances in our understanding of malignant gliomas and progress in treating them, the mechanisms underlying GBM pathogenesis and poor response to conventional therapy are yet unclear.

GSK-3β is a serine/threonine kinase which activity is regulated by site-specific phosphorylation. Full activity of this enzyme generally requires phosphorylation at Tyr-216, and conversely, phosphorylation at Ser-9 inhibits GSK-3β activity. Different studies have shown that GSK-3β is involved in many biological processes, including cell cycle progression, apoptosis and viability, cytoskeletal organization, cellular metabolism and tumorigenesis [4], [5]. Several of these pathways, are implicated in disease pathogenesis, which has prompted efforts to develop GSK-3β inhibitors for therapeutic applications. GSK-3β plays an important role in glucose metabolism and it is thought to facilitate the development of non-insulin-dependent diabetes [6]. Also, GSK-3β has an important role in promoting inflammatory processes through its activation of the transcription factor NF-κB [7]. This kinase has also been implicated in the development of Alzheimer disease and other neurodegenerative disorders [8]. Lastly, several studies have identified a specific role for GSK-3β on proliferation and apoptosis of cancer cells. GSK-3β activation has been associated with prostate cancer progression [9], and inactivation of this enzyme activates a p53-dependent apoptosis pathway resulting in a diminished colorectal cancer cell growth [10].

The thiadiazolidinone compound TDZD-8 belongs to a family of molecules, which was originally described as non-ATP competitive inhibitors of glycogen synthase kinase 3β (GSK-3β) [11], [12]. In line with the implication of GSK-3β-activated pathways in disease pathogenesis, TDZD-8 has been shown to be a protective agent in multiple murine models of disease such as arthritis, spinal cord injury, colitis, and septic shock [13], [14], [15], [16], [17]. More recently, TDZD-8 has been shown to selectively induce death of several major forms of leukemia cells, including malignant myeloid stem and progenitor populations, while sparing normal hematopoietic tissue [18].

In an effort to expand strategies for targeting glioblastoma cells, we have explored the effects of TDZD-8 on glioblastoma development. We demonstrate that TDZD-8 is a potent anti-proliferative and pro-apoptotic agent of glioma cells *in vitro* and *in vivo*. These effects are associated with an early activation of extracellular signal-regulated kinase (ERK), which is followed by an increased expression of the early growth response-1 (EGR-1) and p21. TDZD-8 also elicited a sustained activation of ERK which lead to a phosphorylation of p90RSK and a concomitant inhibition of GSK-3β through phosphorylation of Ser-9. We also demonstrate that TDZD-8 inhibits the growth and neurosphere formation and self-renewal capacity of GL261 cells. As such, these findings identify TDZD-8 as a potential therapeutic agent for the treatment of high grade gliomas.

## Results

### TDZD-8 treatment reduces glioblastoma development *in vivo*

**Figure 1:**
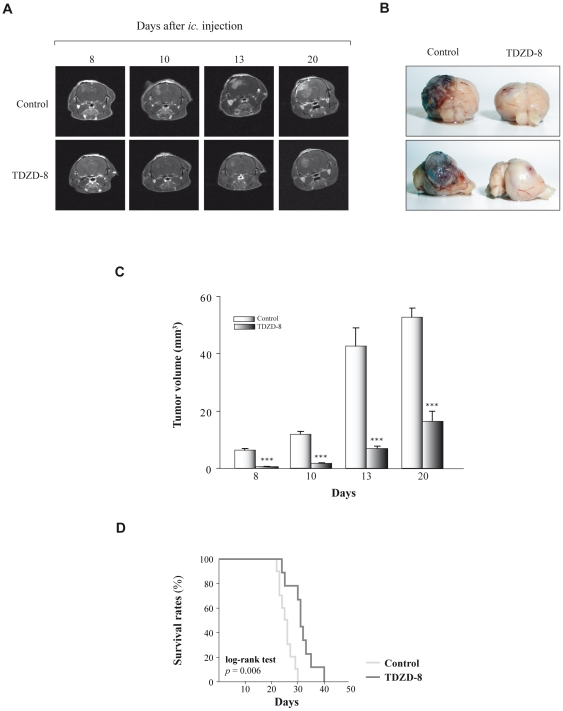
Effects of TDZD-8 treatment on tumor growth *in vivo*. (A) Representative T_1_ magnetic resonance imaging (MRI) pictures obtained form mice injected with GL261 and treated with TDZD-8 (5 mg/Kg). T_1_-weighted imaging was performed at 7 Tesla as described in Materials and Methods at different times after injection. (B) Representative photographs of GL261 tumors 24 days after implantation are shown. (C) Quantitative analysis of total tumor volumes. Values represent the mean ± SD from five different animals. (D) Kaplan-Meier plots and log-rank statistics analysis of overall survival reveal that TDZD-8 treatment significantly improves survival of tumor-bearing mice compared with their non-treated controls (log-rank test *p* = 0.006).

To analyze the effects of TDZD-8 on glioblastoma growth *in vivo*, we orthotopically implanted GL261 glioma cells into adult mice brains to generate tumors. The murine glioma GL261 model has been the most common used syngeneic transplant model for both subcutaneous and intracranial experimental glioma tumors [19], [20], [21]. This particular intracranial animal model recapitulates many of the histopathological and biological features of human glioma including necrosis with pseudopalisading, blood vessels infiltration and presence of giant multinucleated cells [22]. First, we monitored tumor growth *in vivo* by magnetic resonance imaging (MRI) at different times after implantation. Animals treated with TDZD-8 1 day after GL261 cell implantation showed a delayed onset and progression of tumors compared to control animals (Fig. 1A, B). Also, tumor volume, as assessed by T_1_-weighed images after gadolinium contrast administration, was significantly reduced in mice treated with TDZD-8 (Fig. 1A). About 84% reduction in tumor volume was observed in tumors derived from TDZD-8-treated animals at 13 days post-injection (Fig. 1C). This strong reduction in the tumor growth potential induced by the compound was also observed 20 days post-injection. Both the log-rank test and Kaplan-Meier analysis of the survival data demonstrated a significant survival advantage for the mice treated with TDZD-8 when compared to their controls (40 *versus* 30 days) (Fig. 1D). Log-rank analysis of the data yielded a *p* value of 0.006. Of note, this delayed tumor growth was also detected when the TDZD-8 treatment was started 6 days after GL261 cell injection (Fig. S1).

**Figure 2:**
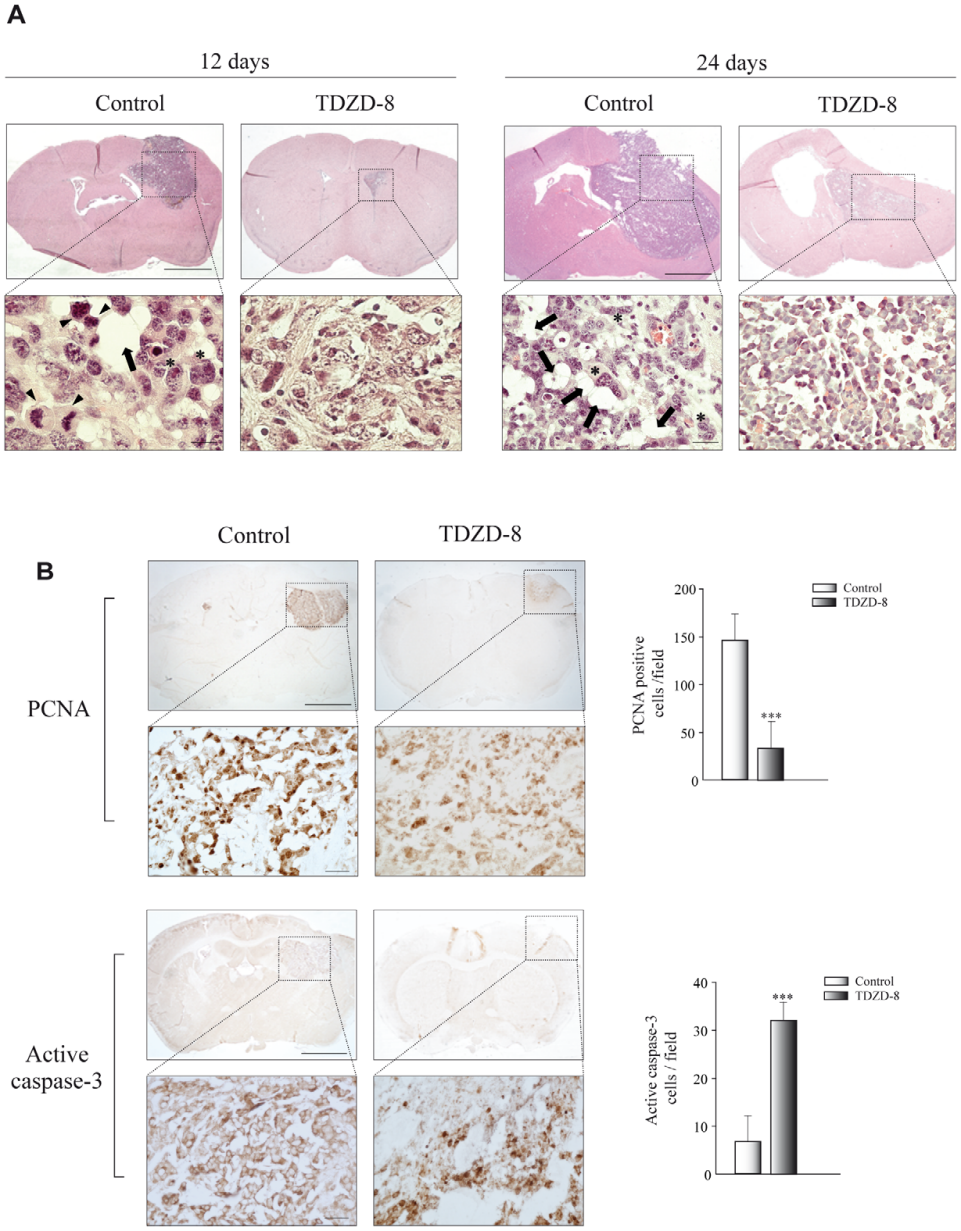
Histological and immunohistochemical analysis of tumors induced by GL261 glioblastoma cells. (A) Representative hematoxylin-eosin stained sections of tumors at 12 and 24 days after injection. Insets show higher magnifications of images shown in the upper panels. Tumors derived from control animals showed clear mixoid matrices (arrows), multinucleated malignant and pleomorphic cells (asterisks), and numerous mitotic figures (arrowheads). (B) Immunohistochemical study of tumor sections for PCNA and active caspase-3 detection 12 days after injection of GL261 cells. Insets show a higher magnification of the images shown in the upper panels. Scale bars, 300 µm. Insets scale bars, 25 µm. Quantification of the number of active caspase-3 and PCNA positive cells was evaluated in tumor sections and values expressed as mean ± SD. positive cells/field. ***p≤0.001.

We next performed histological examination of tumor tissues 12 and 24 days after treatment (Fig. 2A). Microscopically all the tumors were made of sheets of malignant cells that leaved some microcysts among them. These cells showed highly atypical nuclei with prominent nucleoli and an eosinophilic cytoplasm with filamentous elongations. Many multinucleated malignant cells were also seen (Fig. 2A, asterisks in insets). Altogether these features are characteristic of a typical human high-grade glioma. The grade of pleomorphism and nuclear atipia and the mitotic activity were reminiscent of that found in human glioblastoma multiforme. Tumors from control animals presented more mixoid matrices (Fig. 2A, arrows in insets) and a higher mitotic activity with more than 5 mitotic figures per high power field (Fig. 2A, arrowheads in insets), as compared with TDZD-8-treated animals.

To further understanding on tumors characteristics we performed PCNA and active caspase-3 immunohistochemistry 12 days after implantation to assess the effect of TDZD-8 on proliferation and apoptosis. Quantification of the data revealed that TDZD-8-treated animals showed a significant reduction of PCNA expression (Fig. 2B), suggesting a growth-suppressing action of this compound *in vivo*. Figure 2B also shows that mice treated during 12 days with TDZD-8 presented tumors with elevated active caspase-3 expression, compared with vehicle-treated animals, indicating that TDZD-8 promoted apoptosis of glioma cells *in vivo*.

### TZDZ-8 decreases cell proliferation and survival *in vitro*

**Figure 3:**
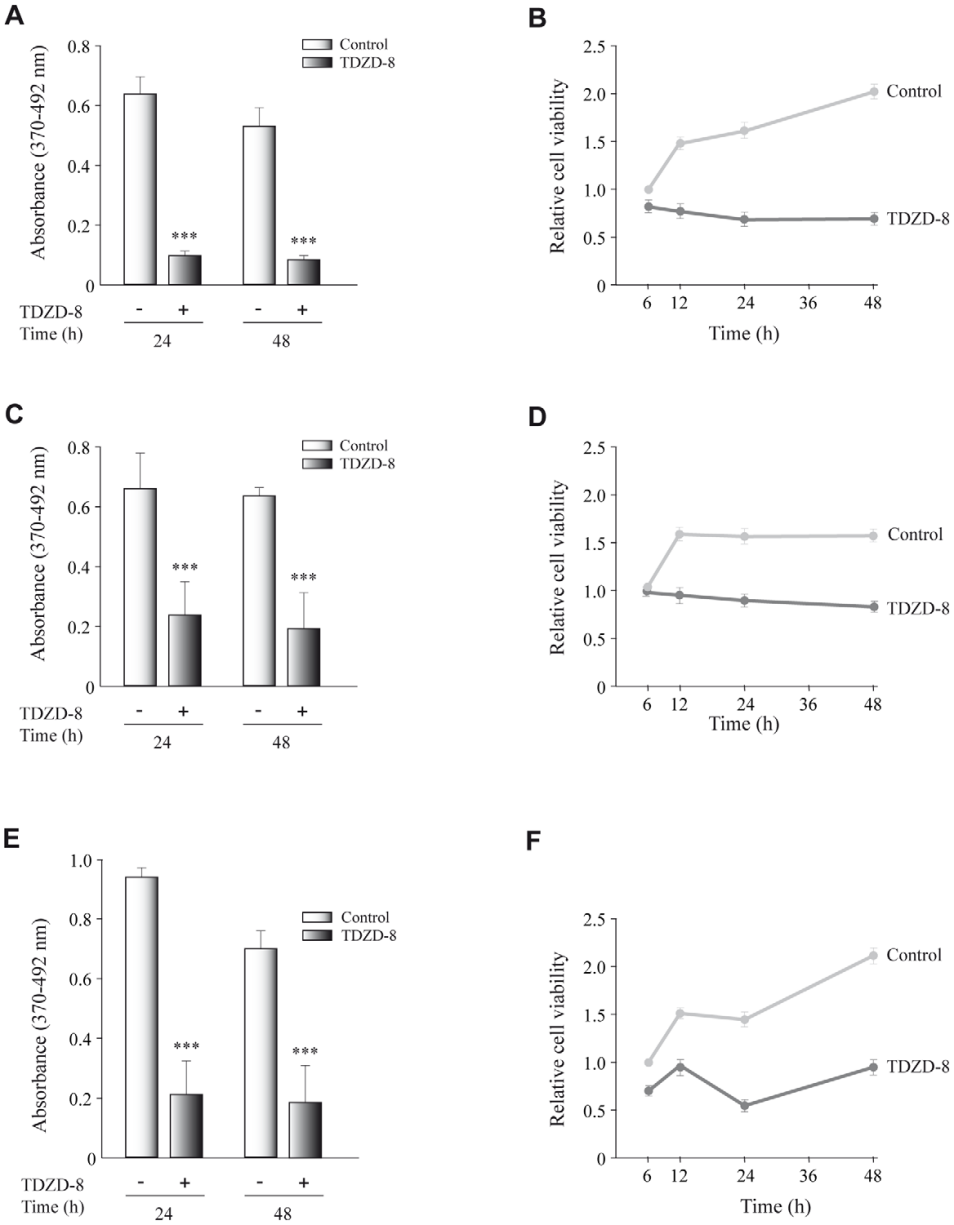
Effect of TDZD-8 treatment on cell proliferation and survival in different glioblastoma cell lines. GL261 (A, B), A172 (C, D) and U373 (E, F). (A, C, E) Proliferation rates determined by a BrdU incorporation assay. Tumoral cells were seeded into individual wells of a 96-well plate and cultivated for 24 and 48 h in the presence or absence of TDZD-8 after which BrdU was added to the culture medium. Cells were harvested 16 h after BrdU addition. Indicated are the means ± SD measured. (B, D, F) Cells were seeded in a 96-well plate and at different times after plating cell viability was determined by the MTT assay, as indicated in Materials and Methods. Values are the means ± SD of at least three different experiments.

To understand the mechanism by which TDZD-8 inhibits tumor growth, we initially tested its antiproliferative effect on GL261 glioblastoma cells analyzing BrdU incorporation. Exponentially growing GL261 cells were exposed to 20 µM TDZD-8 for 24 and 48 h, and their proliferation was monitored. A significant decreased in proliferation was observed in cells treated for both 24 and 48 h with 20 µM TDZD-8 compared with untreated control cells (Fig. 3A). Additionally, cell viability, measured by the MTT assay, was significantly diminished in TDZD-8-treated cells (Fig. 3B), compared to controls. Thus, TDZD-8 has an antiproliferative effect on GL261 glioblastoma cells. This anti-proliferative effect of TDZD-8 was also observed in other two human glioblastoma cell lines, A172 (Fig. 3C, D) and U373 (Fig. 3E, F).

**Figure 4:**
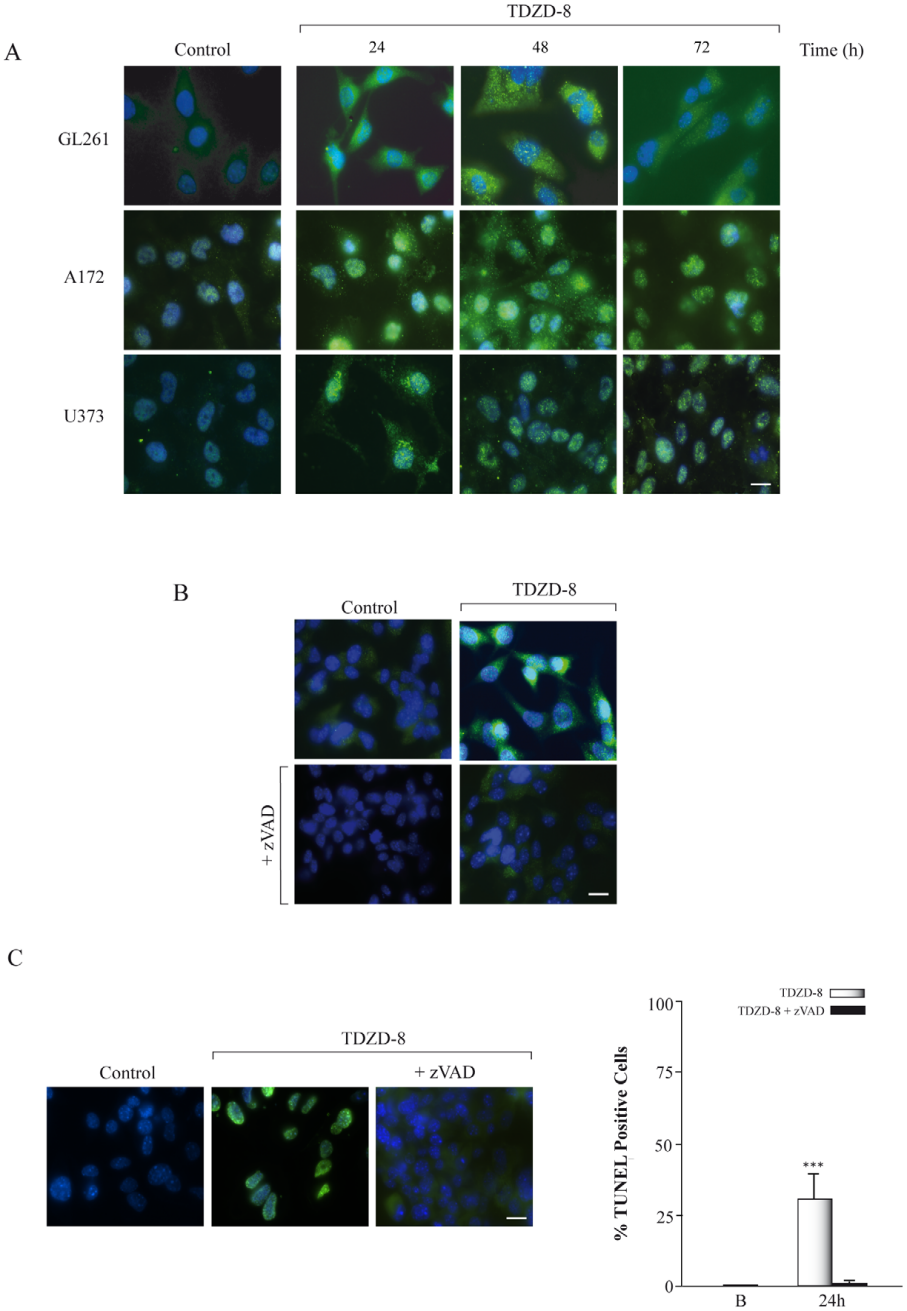
Effect of TDZD-8 treatment on apoptosis. (A) Different glioblastoma cell lines were grown on glass coverslips, treated with TDZD-8 for different times and active caspase-3 was analyzed by immunofluorescence using a specific anti-active caspase-3 antibody. (B) GL261 glioblastoma cells were grown for 24 h on glass coverslips, treated with TDZD-8 or TDZD-8 plus zVAD-fmk and active caspase-3 was analyzed by immunofluorescence using a specific anti-active caspase-3 antibody. Scale bar, 25 µm. (C) GL261 glioblastoma cells were grown for 24 h on glass coverslips, treated with TDZD-8 or TDZD-8 plus zVAD-fmk and TUNEL analysis was performed. Representative immunofluorescence images are shown. Scale bar, 25 µm. Quantification of TUNEL-positive cells 24 h after TDZD-8 treatment is shown in the right panel. B, basal non-treated cells. Values are the means ± SD of at least three different experiments.

To further investigate the role of TDZD-8 in cell growth inhibition, cell death was evaluated by active caspase-3 and TUNEL analysis (Fig. 4). Treatment of GL261 cells at different times with TDZD-8 resulted in an increase of apoptosis that was evident by an increase in the abundance of cleaved caspase-3 (Fig. 4A). Yet again, this proapoptotic effect was also observed in the A172 and U373 glioma cell lines. TUNEL analysis of GL261 cells showed a significant increase in the number of apoptotic cells after TDZD8 treatment (Fig. 4C). Addition of the caspase inhibitor N-benzoylcarbanyl-Val-Ala-Asp-fluoro methylketone (zVAD-fmk) inhibited TDZD-8-induced caspase activation and cell death (Fig. 4B, C). Altogether, these results indicate that TDZD-8 treatment led to a growth arrest of glioblastoma cells by a diminution of cell proliferation and an induction of apoptosis.

### Effects of TDZD-8 on the mitogen-activated protein kinase (MAPK) pathway and NF-κB activation

**Figure 5:**
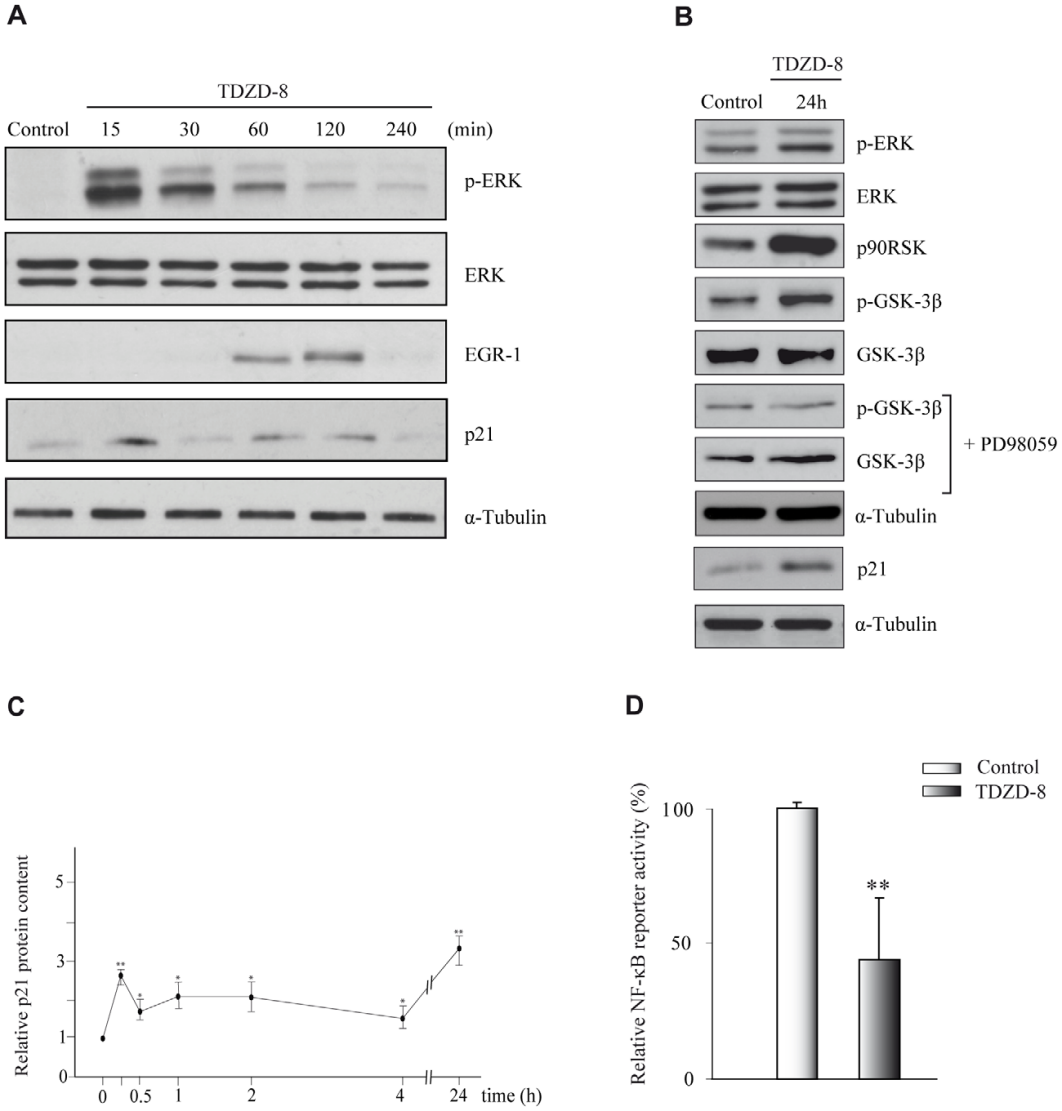
TDZD-8 activates the ERK signaling pathway. (A, B) GL261 cells were treated or not with TDZD-8 for different times and then stained with the corresponding primary antibodies. Some cultures were pretreated for 1 h with the ERK inhibitor PD98059. (C) Quantification of the kinetic induction of p21 by TDZD8. (D) A reporter assay was performed to measure the transcriptional activity of NF-κB. GL261 cells were transfected with 3xNF-tk-luc reporter construct for 48 h in the presence or absence of TDZD-8 and luciferase activity was measured.

We next investigated possible signaling pathways involved in the TDZD-8 anti-proliferative effects on glioblastoma cells. Activation of ERK has been associated with a diminution of cell survival in different tumor cell lines, including glioblastoma cell lines [23], [24], [25]. Therefore, we next investigated the possible involvement of this pathway in the anti-proliferative and pro-apoptotic effects of TDZD-8 described here. First, we evaluated the phosphorylation status of ERK1/2. As can be seen in Figure 5A, ERK1/2 become rapidly phosphorylated after TDZD-8 treatment. Next, we examined the expression of two genes known to play an important role in cell growth and which are downstream targets of the ERK cascade [26], the early growth response -1 (EGR-1) gene and p21. An increase in the expression of both genes was observed 1 and 2 hours after TDZD-8 addition to GL261 cells (Fig. 5A). In addition to this rapid effect, we also detected a sustained activation of ERK1/2 and a sustained increase of p21 (Fig. 5B, C). Since the ribosomal S6 kinase (p90RSK) is a well-known target of ERK [27], [28] and activated p90RSK can inactivate GSK-3β by phosphorylation at Ser9, we then analyzed the effect of TDZD-8 treatment on the phosphorylation status of these enzymes. As shown in Figure 5B an increase in phosphorylation of p90RSK at Ser380 and GSK-3β at Ser9 was detected 24 hours after TDZD-8 treatment. Noteworthy, although the increase in phosphorylation of ERK1/2 is not as high as the one observed at shorter time points (Fig. 5A) this enhancement is consistent and sufficient to initiate the signaling cascade leading to activation of their target p90RSK and subsequent inactivation of GSK-3β. To further substantiate the notion that the ERK/p90RSK pathway is involved in the phosphorylation of GSK-3β after TDZD-8 treatment, we used the PD98059 inhibitor to block this pathway. Our results show that addition of PD98059 significantly inhibited the phosphorylation of GSK-3β at Ser9, suggesting that activation of the ERK pathway plays a major role in the phosphorylation and concomitant inactivation of this enzyme [29].

Since it has been also shown that GSK-3β regulates tumor cell survival through a NF-κB-dependent pathway [7], [30] we next tested whether GSK-3β inhibition by TDZD-8 could affect NF-κB activity in GL261 cells. To this end, we performed transient transfection assays using a reporter construct that contains three copies of the consensus NF-κB response element (3xKBtk-luc) to determine NF-κB activity in control and TDZD-8-treated cells. As shown in Figure 5D, TDZD-8 inhibited NF-κB activity, which is in accordance with previously published data [31], [32].

### Effect of TDZD-8 on glioblastoma stem cells

**Figure 6:**
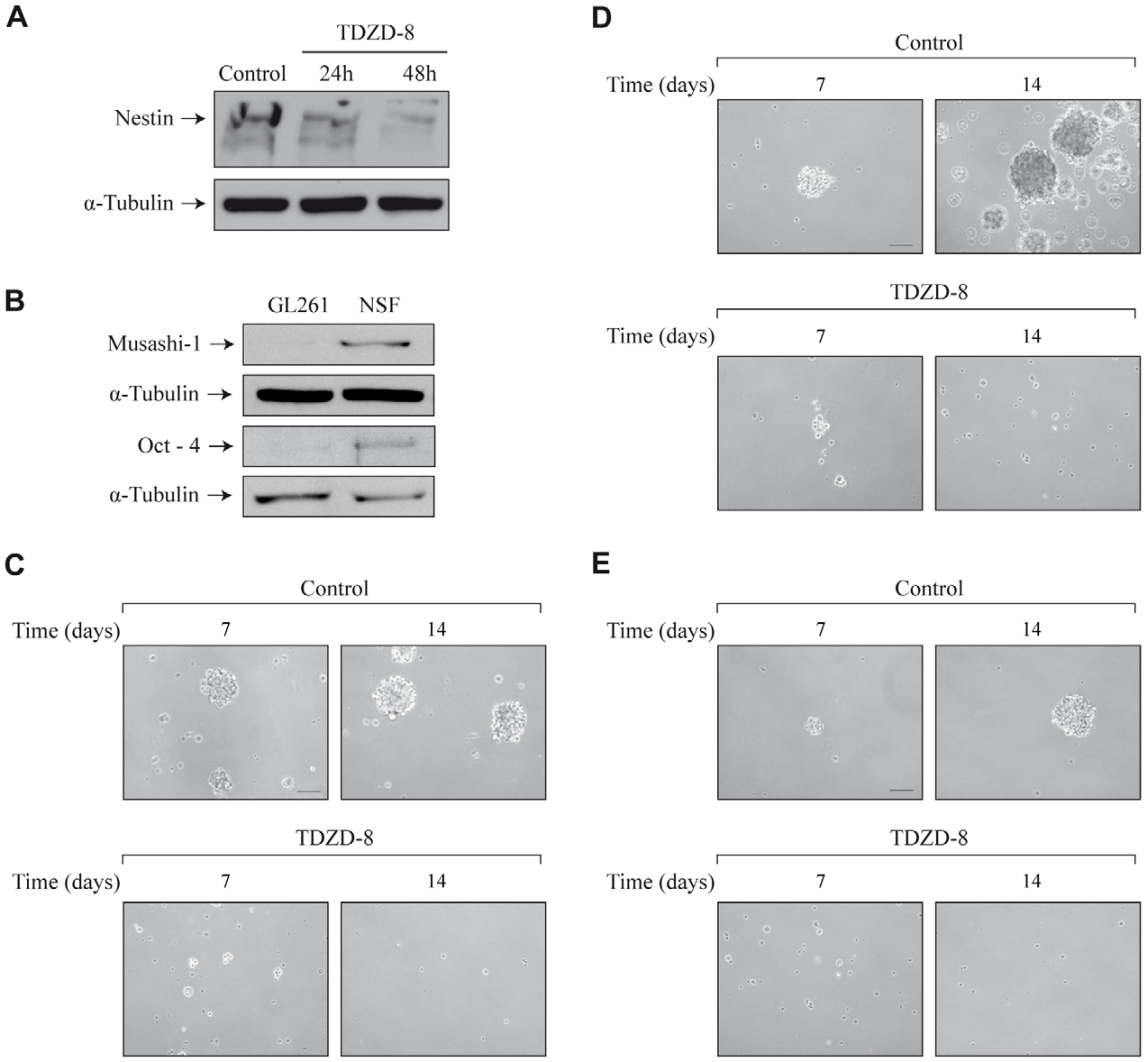
Effect of TDZD-8 on neurosphere formation. (A) Representative Western blot showing Nestin expression in adherent GL261 cells treated with TDZD-8. (B) Representative Western blot of stem cells markers musashi-1 and oct-4 in adherent GL261 and neurospheres cultures 7 days after plating. (C) Representative microphotographs of primary neurosphere cultures 7 and 14 days after plating in neurosphere medium. D) Primary neurospheres were dissociated and plated to analyze secondary neurosphere formation. Representative microphotographs are shown. (E) Representative microphotographs showing self-renewal capacity of neurosphere cultures growing in the presence or absence of TDZD-8. Scale bar, 100 µm.

The emerging cancer stem cell model suggests that tumors are organized in a hierarchy with a subpopulation of cancer stem cells (CSCs) responsible for tumor maintenance and progression. Therefore, we determined whether TDZD-8 could also exert an antiproliferative effect on progenitor glioblastoma stem cells by analyzing its effect in GBM-derived neurospheres. It has been shown that the major intermediate filament protein Nestin could be used as a marker of brain CSCs as well as normal neural stem and precursor cells [33], [34], [35], [36]. Therefore, as a first step to establish if TDZD-8 could affect the CSC population in GL261 glioblastoma cells, we examined its effect on the expression of Nestin in adherent cultures of GL261 cells. The results shown in Figure 6A reveal a significant reduction in the protein levels of Nestin in those cultures treated with TDZD-8, which could indicate a loss of stem cells induced by this compound. To further elucidate this point, we examined the effects of TDZD-8 on the formation and growth of GL261-derived neurospheres (primary neurospheres). First, we analyzed the levels of two well-known stem cell markers such as musashi-1 and oct-4 in adherent GL261 cells and neurosphere cultures. As shown in Figure 6B an increased amount of these proteins was observed in the neurospheres indicating an increased population of stem cells in these cultures, compared with attached GL261 cells. Next, we studied if TDZD-8 could affect the CSC population in GL261 glioblastoma cells. Figure 6C shows that TDZD-8 inhibited the formation of primary neurospheres. We observed a significant decrease, at both 7 and 14 days of culture, in the number and volume of TDZD-8-treated neurospheres, compared with controls. To more stringently test the effect of TDZD-8 on the ability of GBM cells to generate new spheres, actively growing 7 day-old GL261-derived primary neurosphere cultures were dissociated, and equal numbers of viable cells were replated in fresh neurosphere medium to generate new neurospheres (secondary neurospheres). Treatment of these cultures with TDZD-8 almost completely blocked the formation of secondary neurospheres (Fig. 6D). Additionally, when primary TDZD-8-treated cultures were dissociated and cultured again for 7 days in the absence of TDZD-8, no formation of secondary neurospheres was observed (data not shown). Finally, to test for the effect of TDZD-8 on the self-renewal of the neurosphere cultures, we dissociated established 7 day-old primary neurosphere cultures, plated them at a very low density [37], and analyzed the capacity to form secondary spheres. As shown in Figure 6E cultures treated with TDZD-8 did not give rise to secondary neurospheres 7 and 14 days after plating, indicating that these cultures did not contain self-renewing stem cells.

## Discussion

In this study, we show for the first time that TDZD-8 suppresses the growth of glioma tumors *in vivo* and exerts anti-proliferative and pro-apoptotic activities in glioma cells *in vitro*. These effects were accompanied by an activation of the ERK/p90RSK pathway, a concomitant phosphorylation and inactivation of GSK-3β, and an inhibition of NF-κB activity. Finally, our data showing that TDZD-8 decreases the formation of neurospheres, both at low and high cell density, suggest that this drug could have an inhibitory effect on glioblastoma stem cells proliferation and self-renewal. Collectively, our findings suggest that TDZD-8 might be of therapeutic importance for the treatment of high-grade gliomas.

Previous studies have implicated GSK-3β in tumorigenesis. GSK-3β activation has been associated with prostate cancer progression and TDZD-8 has an inhibitory effect in these tumor cells [9]. Also, GSK-3β inactivation induces a p53-dependent apoptotic pathway resulting in a diminished colorectal cancer cell growth [10], [38]. More recently, it has been shown that TDZD-8 inhibits proliferation and induces death of myeloma cell lines [39] and of malignant myeloid progenitors, while sparing normal hematopoietic tissue [18]. In the case of tumors from the central nervous system, contradictory results have been reported. Kotliarova et al. using different glioma cell lines have shown that GSK-3β activation promotes cell survival [30], whereas Ma et al. [40] have shown that GSK-3β activation is required for the induction of apoptosis in SK-N-MC neuroblastoma cells. Our data are in agreement with the results obtained by Kotliarova et al, here we clearly show that the inactivation of GSK-3β by TDZD-8 inhibits glioblastoma tumor growth in vivo. Thus, tumor development was significantly delayed and animal survival improved (*p* = 0.006) in mice injected with this compound. Moreover, TDZD-8 tumors lacked the aggressiveness of control tumors, including necrotic foci and a higher mitotic activity.

In view of the pleiotropic effects of TDZD-8, it is very likely that different mechanisms can be underlying its action. Initially TDZD-8 was synthesized as a high affinity ATP-non-competitive inhibitor of GSK-3β [11], [12]. However, recently, several lines of evidence indicate that a GSK-3β-independent mechanism may be involved in TDZD-8 actions. Guzman et al have suggested that the activity of TDZD-8 can be independent of this inhibition since other known GSK-3β inhibitors fail to induce leukemia-specific cell death [18]. In this regard, we have shown that other TDZD compounds exert anti-inflammatory and neuroprotective effects in the brain through activation of the nuclear receptor PPARγ [41], [42]. Here, we show that TDZD-8 rapidly activates ERK signaling pathway, which promotes EGR1 expression and an increase in p21 levels. Given the fact that this activation is very fast, it is very unlikely that it could be mediated trough activation of PPARγ. Our findings further corroborate other studies showing that an activation of MAPK signaling pathway in U-87MG glioma cells leads to a proliferation arrest mediated by an increase in EGR-1 expression which concomitantly stimulates p21 transcription [26]. In addition, we also show a delayed activation of ERKs in response to TDZD-8, which is followed by a phosphorylation and activation of p90RSK, a well-known target of these kinases [27], [28]. This activation results in a phosphorylation and inactivation of GSK-3β [29]. These results are in agreement with previously published data showing that an activation of ERK is associated with a diminution of cell survival in different tumor cell lines, including glioblastoma cell lines [23], [24], [25]. Our results therefore suggest that TDZD-8 can inhibit GSK-3β activity not only by directly interacting with this enzyme but also through its phosphorylation at Ser9 via MAPK pathway activation.

It has been reported that inhibition of GSK-3β by different compounds, including TDZD-8, causes an inactivation of NF-κB activity [31], [32] and Kotliarova et al have shown that several small molecular inhibitors of GSK-3β activity inhibit glioma cell survival in part through a decrease in intracellular NF-κB activity [30]. Consistent with these data we also show here that treatment of GL261 glioma cells with TDZD-8 led to a decrease in NF-κB activity within these cells. In this regard, other groups have shown that survival of different tumor cells depends on GSK-3β activity trough a NF-κB-dependent pathway [7], [43], [44].

There is mounting evidence that neural stem cells can be transformed into cancer stem cells and give rise to malignant gliomas by escaping the mechanisms that control proliferation and programmed differentiation [45], [46], [47]. Several data implicate glioma stem cells in tumor maintenance and therapeutic resistance [48], [49], [50], in consequence the discovery of putative brain tumor stem cells identifies a new cellular target that might be susceptible to novel treatments. Our results suggest that, in addition to have an inhibitory effect upon the bulk of glioblastomas, TDZD-8 could also inhibit cancer stem cell growth. Treatment with TDZD-8 resulted in an inhibition of neurosphere formation in culture. TDZD-8 inhibited the proliferation and expansion of these neurospheres and hampered their capacity of self-renewal. One feature that contributes to the ability of a stem cell to survive is its inherent resistance to drugs; in this regard our results are particularly important since they suggest that TDZD-8 could reduce the tumor-initiating cells. Our results provide compelling evidence that TDZD-8 is able to both inhibit the bulk of the tumor, characterized by actively cycling cells, and to hinder the growth of neural stem cells characterized by a low rate of division.

In summary, we have presented here the first evidence that TDZD-8 inhibits gliomagenesis and targets glioma stem-like cells and thus may hold promise for treatment of human gliomas.

## Materials and Methods

### Animal Experiments

Adult male C57BL/6 mice (*n* = 10 per group) were anaesthetized by intraperitoneal injection of ketamine (60 mg/Kg) and medetomidine (0.125 mg/Kg) and positioned in a stereotaxic apparatus (Kopf Instruments, CA). To establish intracranial tumors GL261 cells (125,000 cells) were implanted unilaterally into the right hemisphere using the following coordinates from Bregma: posterior −1.06 mm; lateral 3 mm and a depth of 3 mm, according to the atlas of Paxinos and Franklin [51]. The mice were then housed individually to recover. One day after implantation of GL261 cells, two groups of mice were injected daily intraperitoneally with 5 mg/Kg of TDZD-8 (Sigma) or DMSO (control group) during 22 days. In other group of animals, treatment with TDZD-8 was started 6 days after implantation of GL261 cells, during 7 days. All procedures with animals were specifically approved by the ‘Ethics Committee for Animal Experimentation’ of the Instituto de Investigaciones Biomedicas (CSIC-UAM), permit number PN 2007/108, and carried out in accordance with the protocols issued which followed National (normative 1201/2005) and International recommendations (normative 86/609 from the European Communities Council). Special care was taken to minimize animal suffering.

### Magnetic Resonance Imaging

Magnetic Resonance Imaging (MRI) was performed using an MRI scanner (Bruker PharmaScan 7.0T, 16 cm; Bruker Medical Gmbh, Ettlingen, Germany). Mice brain MRI was performed with a 90 mm gradient insert and a concentrical 38 mm birdcage resonator, using Paravision v4.0 software (Bruker Medical Gmbh, Ettlingen, Germany) as implemented in a Hewlett-Packard console, operating on a Linux platform. MRI examinations used adult male C57BL/6 mice (*n*≥10 per group) anaesthetized through a plastic mask with 2% isofluorane in 99.9% O_2_. Animals were allowed to breath spontaneously during the experiment and were placed in a heated cradle to maintain the core body temperature at approx. 37°C. The physiological state of the animal was monitored throughout MRI acquisition through the respiratory rate using a Biotrig physiological monitor (Brucker). Gadolinium-DTPA-enhanced T_1_-weighted spin-echo images were acquired at 8, 10, 13 and 20 days after injection with a Rapid Acquisition with Relaxation Enhancement (RARE) [52] sequence in axial orientations (TR: 350 ms, TE: 10.6 ms, averages: 4, FOV: 2.30 cm, acquisition matrix: 256×256, slice thickness: 1.00 mm, number of slices: 16). The *in vivo* spectroscopy protocol acquired two 3x3x3 mm voxels in the striatal area, using a Point-Resolved Spatially Spectroscopy (PRESS) [53] protocol, combined with VAPOR water suppression, [54](TR: 3000 ms, TE: 35 ms, averages: 128). Tumor area was calculated from T_1_-weighted images using Image J Software. Tumor volume was estimated from the summation of tumor areas on each slice, multiplied by slice thickness. Average lesion volume was calculated for each condition.

### Histology and Immunohistochemistry

Brains were dissected and embedded in paraffin. Sections of 10 µm thickness were prepared and stained with haematoxylin and eosin. Paraffin embedded sections, were also used for detecting proliferation and apoptosis in tumors. First, sections were deparaffinized in xylene and rehydrated in graded concentrations of ethanol. Endogenous peroxidase activity was blocked by incubation in H_2_O_2_ and after several rinses in PBS, antigen retrieval was performed by microwaving slides in citrate buffer. Once non-specific binding sites were blocked for 1 hour at room temperature, sections were incubated in humid chamber at 4°C overnight with anti-active caspase-3 (1∶200, R&D Systems) and anti-PCNA (1∶50, Signet Laboratories). After several rinses, sections were incubated for 1 h with a biotinylated secondary antibody and finally processed following the avidin-biotin protocol (Vectastain ABC kit; Vector Laboratories). Tissues were mounted onto gelatin-coated slides, dehydrated, cleared in xylene, and mounted with DePeX (Serva, Heidelberg, Germany). The slides were examined under a Zeiss (Oberkochen, Germany) Axiophot microscope, equipped with an Olympus Optical (Tokyo, Japan) DP-50 digital camera, and a Leica (Nussloch, Germany) MZ6 modular stereomicroscope. For the quantification of active caspase-3 and PCNA expression, the number of positive cells was quantified in 20 random fields at x400 magnification. Data were expressed as mean ± SD positive cells/field.

### Cell culture and treatment

GL261 murine glioblastoma cells were obtained from the NCI-Frederick Cancer Research Tumor Repository (Frederick, MD) and propagated in RPMI medium with 10% fetal bovine serum as described [55]. A172 and U373 human glioblastoma cell lines were obtained from Dr. Manuel Guzman (Complutense University, Madrid, Spain) and propagated in DMEM with 10% FBS. On attaining semiconfluence, and based in dose-response analysis (see Fig. S2), cells were treated with 20 µM TDZD-8, a dose widely used in the literature [18], [56], for different time intervals. Following treatment cells were processed for western blot and immunocytochemical analysis.

For neurosphere formation GL261 cells were plated and grown in regular medium (RPMI, 10% FBS, glutamine, gentamicine and fungizone). Two days after plating, supernatant was collected and replated in a defined serum-free tumor sphere medium formed by Ham's F-12/Dulbecco's modified Eagle's medium (1∶1) supplemented with B27 (Invitrogen, Carlsbad, CA), 20 ng/ml epidermal growth factor (EGF, Peprotech, EC) and 20 ng/ml fibroblast growth factor (FGF, Peprotech, EC). After 1 week in culture some primary neurosphere cultures were treated with TDZD-8 (10 µM) for another 1 week. These primary neurospheres were then dissociated, and 50,000 cells/ml were replated in proliferative conditions, in the absence or presence of TDZD-8, for another 7 or 14 days to score the number of secondary neurospheres generated. For self-renewing experiments, primary neurospheres were dissociated and plated at a density of 2,000 cells/ml for another 7 or 14 days in proliferative medium containing or not TDZD-8. These assays were repeated at least three times in triplicate.

### Proliferation assays

The effect of TDZD-8 on cell proliferation was determined using the non-radioactive BrdU-based cell proliferation assay (Roche) according to the manufacturer's protocol. Cells were seeded in triplicate onto 96-well plates at a density of 2,000 cells/well. After 24 h of growth, cells were treated with 20 µM TDZD-8, 16 h later 10 µM BrdU was added and cells were cultured for another 16 h. BrdU incorporation into the DNA was determined by measuring the absorbance at both 370 and 492 nm on an ELISA plate reader.

Cell viability was measured using the MTT assay (Roche Diagnostic, GmbH), based on the ability of viable cells to reduce yellow MTT to blue formazan. Briefly, cells were cultured in 96-well microlitre plates for various periods of time in the presence or absence of 20 µM TDZD-8, then cells were incubated with MTT (0.5 mg/ml, 4 h) and subsequently solubilized in 10% SDS/0.01 M HCl for 12 h in the dark. The extent of reduction of MTT was quantified by absorbance measurement at 550 nm according to the manufacturer's protocol.

### Immunoblot analysis

Cultured cells, both adherent and floating cancer stem cells, were harvested and lysed in ice-cold RIPA buffer and equal quantities of total protein were separated by 10% SDS-PAGE. After electrophoresis, proteins were transferred to nitrocellulose membranes (Protran, Whatman, Dassel, Germany) and blots were probed with the indicated primary antibodies, as previously described [57]. The antibodies used were the following: rabbit polyclonal anti-p-ERK1/2 (1∶1000, Cell Signaling), rabbit polyclonal anti-ERK1/2 (Cell Signaling), rabbit polyclonal anti-p90RSK (1∶1000, Cell Signaling), rabbit polyclonal anti-p-GSK-3β (1∶500, Cell Signaling), mouse monoclonal anti-GSK-3β (1∶250, BD Transduction), rabbit polyclonal anti-EGR-1 (1∶1000, Santa Cruz Biotechnology), rabbit polyclonal anti-p21 (1∶1000, Abcam), rabbit polyclonal anti-Musashi 1 (1∶1000, Abcam), rabbit polyclonal anti-Oct-4 (1∶500, Santa Cruz Biotechnology), rabbit polyclonal anti-Nestin (1∶2000; kindly provided by Dr. M. Vallejo, Instituto de Investigaciones Biomédicas, Madrid, Spain), and mouse monoclonal anti-α-tubulin (1∶5000, Sigma). Secondary peroxidase-conjugated donkey anti-rabbit and rabbit anti-mouse antibodies were from Amersham Biosciences (GE Healthcare, Buckinghamshire, England) and Jackson Immunoresearch, respectively.

### Immunocytochemical staining

At the end of the treatment period the cultures, grown on glass cover-slips in 24-well cell culture plates, were washed with PBS and fixed for 30 min with 4% paraformaldehyde at 25°C and permeabilized with 0.1% Triton X-100 for 30 min at 37°C. After 1 h incubation with the corresponding primary antibody: anti-active caspase-3 (1∶500, R&D Systems) cells were washed with PBS and incubated with an Alexa-labeled secondary antibody (Invitrogen, San Diego, CA) for 45 min at 37°C. Images were acquired using a Radiance 2100 confocal microscope (Bio-Rad, Hercules, CA), with a 350 nm diode laser to excite DAPI (4,6,diamidino-2-phenylindole) and a 488-Argon laser to excite Alexa 488. Confocal microscope settings were adjusted to produce the optimum signal-to-noise ratio. To compare fluorescence signals from different preparations, settings were fixed for all samples within the same analysis.

### Determination of apoptotic cells

To calculate the extend of cell death, cells were treated or not with TDZD-8 and TUNEL analysis were performed following the manufacturer's recommendations. Caspase-3 activation was analyzed by immunofluorescence analysis using a specific anti-active caspase-3 antibody. Some cultures were treated with the caspase inhibitor z-VAD-fmk.

### Transient transfections

For transient transfection experiments, semi-confluent GL261 cells were transfected with the 3xNFtk-luc reporter plasmid as previously described [58]. Forty-eight hours after transfection, cells were harvested for luciferase and-β-galactosidase (to determine transfection efficiency) activities by using a reporter assay system (Promega, Madison, WI). Each transient transfection experiment was repeated at least three times in triplicate.

### Statistics

Other than the survival experiments, Student's test was used to analyze statistical differences between the different groups. Survival curves were plotted with Kaplan-Meier method and survival for the two groups of animals was studied using log-rank test. Differences were considered statistically significant at *p*<0.05.

## Supporting Information

Figure S1 Effects of TDZD-8 administered after tumor is established. Representative T_1_ magnetic resonance imaging (MRI) pictures obtained from mice treated with TDZD-8 from day 6 after GL261 cells injection. T_1_-weighted imaging was performed at 7 Tesla as described in Materials and Methods. The arrow indicates the day the treatment was initiated.
(1.16 MB TIF)

Figure S2 Cell viability in TDZD-8-treated cells. GL261 glioblastoma cells were incubated with various concentrations of TDZD-8 and viability was assessed by the MTT assay, as indicated in Materials and Methods. Values are the means ± SD of at least three different experiments.
(0.07 MB TIF)
