# Supplementary figures and images for: Inhibition of Glioblastoma Growth by the Thiadiazolidinone Compound TDZD-8
Source: PLoS One. 2010 Nov 8;5(11):e13879. doi: 10.1371/journal.pone.0013879 (PMC2975629; doi:10.1371/journal.pone.0013879)

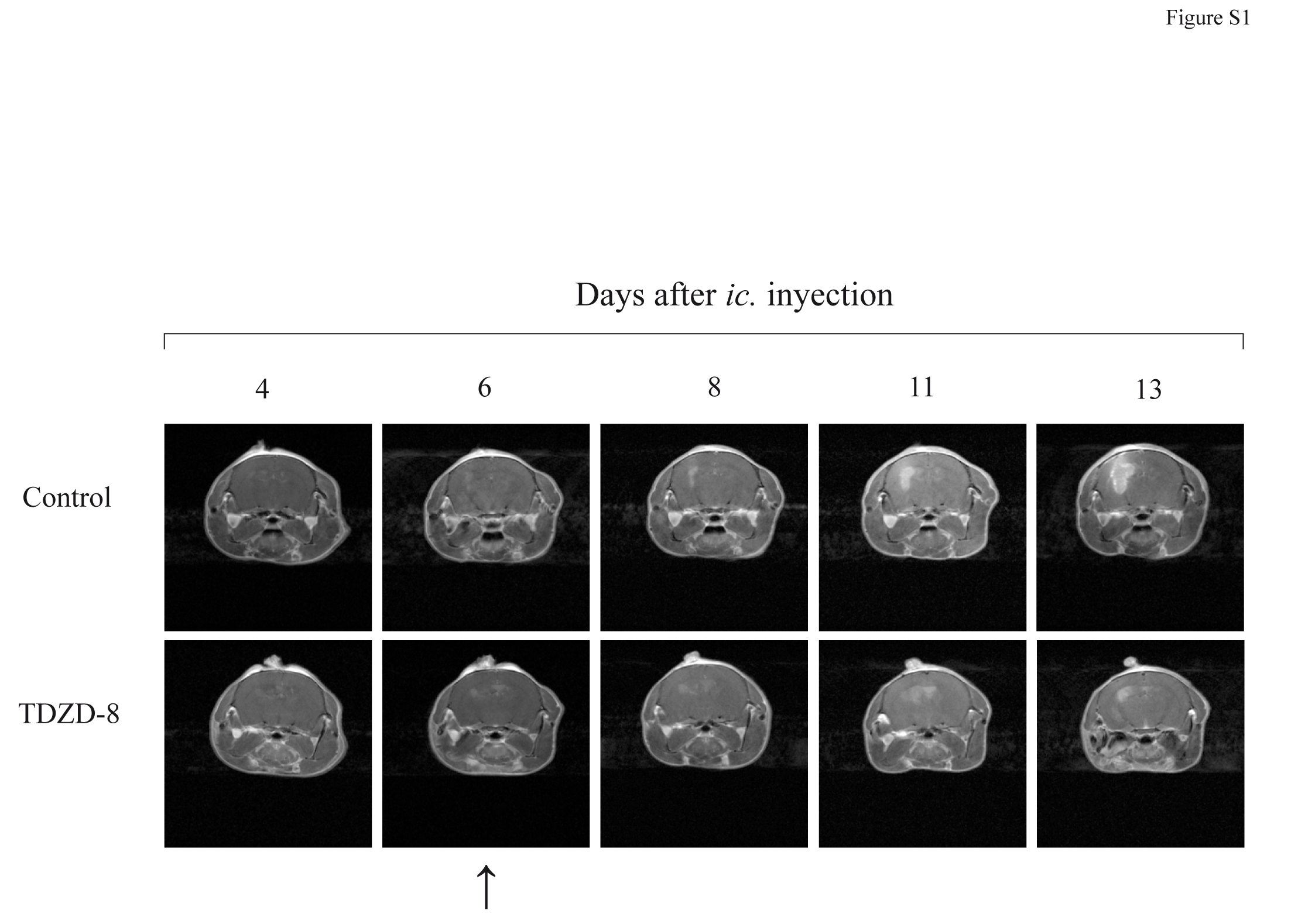

Supplement: Figure S1 — Effects of TDZD-8 administered after tumor is established. Representative T1 magnetic resonance imaging (MRI) pictures obtained from mice treated with TDZD-8 from day 6 after GL261 cells injection. T1-weighted imaging was performed at 7 Tesla as described in Materials and Methods. The arrow indicates the day the treatment was initiated. (1.16 MB TIF) [file pone.0013879.s001.tif]

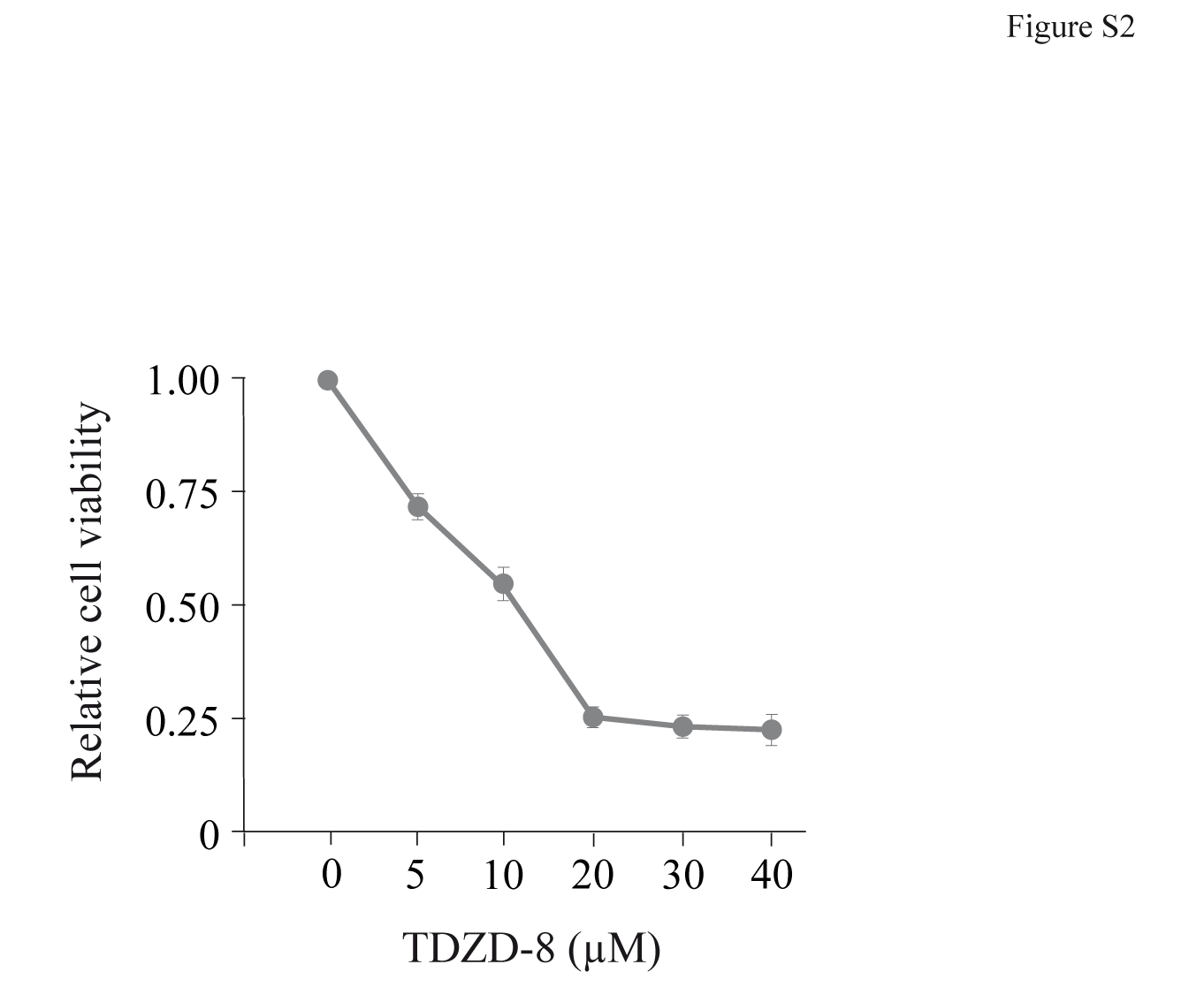

Supplement: Figure S2 — Cell viability in TDZD-8-treated cells. GL261 glioblastoma cells were incubated with various concentrations of TDZD-8 and viability was assessed by the MTT assay, as indicated in Materials and Methods. Values are the means ± SD of at least three different experiments. (0.07 MB TIF) [file pone.0013879.s002.tif]
